# Update on Monoterpenes from Red Macroalgae: Isolation, Analysis, and Bioactivity

**DOI:** 10.3390/md17090537

**Published:** 2019-09-16

**Authors:** Ana-Marija Cikoš, Mladenka Jurin, Rozelindra Čož-Rakovac, Stela Jokić, Igor Jerković

**Affiliations:** 1 Faculty of Food Technology Osijek, University of Josip Juraj Strossmayer in Osijek, 31000 Osijek, Croatia; acikos@ptfos.hr (A.-M.C.); sjokic@ptfos.hr (S.J.); 2 Ruđer Bošković Institute, Bijenička 54, 10000 Zagreb, Croatia; mladenka.jurin@irb.hr (M.J.);; 3 Faculty of Chemistry and Technology, University of Split, R. Boškovića 35, 21000 Split, Croatia

**Keywords:** macroalgae, monoterpenes, biosynthesis, isolation, bioactivity

## Abstract

Macroalgae produce a wide range of monoterpenes as secondary metabolites of mevalonate (MVA) and/or methylerythritol phosphate (MEP) pathway (often including haloperoxidase action). Great biodiversity of macroalgal monoterpenes was reported including acyclic, monocyclic, and bicyclic structures. Halogenated monoterpenes exhibited significant biological activity (e.g., anticancer, antiplasmodial, and insecticidal) that is influenced by the number of present halogens (higher halogen content is preferable, especially bromine) and their position within the monoterpene skeleton. In distinction from the existing reviews, the present review provides novelty with respect to: (a) exclusively monoterpenes from red macroalgae are targeted; (b) biosynthesis, isolation, and analysis, as well as bioactivity of monoterpenes are represented; (c) the methods of their isolation, analysis, and structure elucidation are summarized; (d) the bioactivity of macroalgal monoterpenes is systematically presented with emphasis on anticancer activity; (e) the literature references were updated.

## 1. Introduction

Natural organic compounds exhibited a significant role in the development of new drugs [1] and often serve as a model for making new semi-synthetic and synthetic compounds with improved biological activity [2]. The search for new drugs has led to the testing of naturally occurring compounds from marine environment [3]. Marine macroalgae produce a number of structurally different compounds that exhibit various pharmacological properties [4,5,6], including antiviral, antibacterial, antifungal, cytotoxic [7], insecticidal, antihelmitic, antifeedant, antioxidant, anti-inflammatory [5,7], and antitumor properties [8]. The algae are also source of nutrients [9], containing carbohydrates, amino acids, fatty acids, fibers, vitamins (A, C, B1, B2, B6, and niacin), and minerals (iodine, potassium, magnesium, and calcium). In addition to primary metabolites, algae also produce a large variety of natural organic compounds, which do not play a prominent role in primary metabolism [10]. Such substances are called secondary (specialized) metabolites and are produced in specialized cells. Terpenes (monoterpenes, sesquiterpenes, and diterpenes) are algal and plants specialized metabolites [10,11]. They are a large and structurally diverse group of compounds (isoprenoids) of the general formula (C_5_H_8_)_n_ containing isoprene unit (2-methylbuta-1,3-diene) that are found in the volatile oils from land plants and seaweeds [11]. Marine monoterpenes remained undiscovered until 1955 when seven monoterpenes of green alga *Ulva pertusa* Kjellman were reported [12]. The first unusual marine monoterpenes were not identified until 1973 when the isolation of polyhalogenated compounds from *Aplysia californica* J.G. Cooper was done [13,14]. During the last decades, following these initial discoveries, new marine monoterpenes have been reported including degraded monoterpenes and monoterpenes of mixed biogenesis [15,16,17,18].

This review highlights the occurrence of monoterpenes in macroalgae with emphasis on the cyclic and halogenated monoterpenes since they were the most active. Macroalgal monoterpenes extraction techniques, methods for the analysis, and structure elucidation are presented, as well as their bioactivity. Available literature, including reviews on biochemistry, biosynthesis, and bioactivity have been focused mainly on monoterpenoids from terrestrial plants [19,20,21,22,23,24,25,26]. Several reviews on marine volatile halogenated metabolites (including monoterpenes) exist [7,27,28,29,30,31]. Neither of these reviews represented comprehensively all domains which are presented in this paper. Kladi et al. [27] not only put an emphasis on monoterpenes, but also on other volatile metabolites present in red algae with short description of their bioactivities. Cabrita et al. [28] reported only few halogenated monoterpenes with antiplasmodial activity. Ibrahim et al. [30] in the review mentioned only the insecticidal activity of few monoterpenes. El Gamal [7] covered all bioactive compounds present in the algae and, among them, emphasized monoterpenes as anticancer agents. Monoterpenes were also reviewed with other nutrients and secondary metabolites of the macroalgae [29]. Zatelli et al. [31] represented that brown algae of the genus *Dictyopteris* also contained some monoterpenes but in much lower concentration when compared to red algae. In distinction to the published reviews, the present review presents several novelties: (a) exclusively monoterpenes (acyclic, monocyclic, and bicyclic) from red macroalgae, as the main source of monoterpenes, were targeted; (b) the methods of their isolation, analysis, and structure elucidation were systematically summarized in the tables; (c) the bioactivity (anticancer, antiplasmodial, and insecticidal) of macroalgal monoterpenes was systematically presented; (d) the literature references were updated.

## 2. Biosynthesis of Monoterpenes in Macroalgae

Available literature for the biosynthesis of monoterpenes focus mostly on the algae belonging to the class *Rhodophyta* (red algae). The halogenated monoterpenes were found in the marine algae and they were synthesized mostly for chemical defense from herbivores [32]. Bromine and chlorine ions are abundant in seawater and involved in the formation of halogenated monoterpenes promoted by bromoperoxidase which is present in many red algae species [33]. A wide variety of these compounds was found in three genera of red algae including *Ochtodes*, *Plocamium*, and *Portieria* [34,35]. 

### 2.1. Mevalonate (MVA) Pathway and Methylerithritol Phosphate (MEP) Pathway

**Figure 1:**
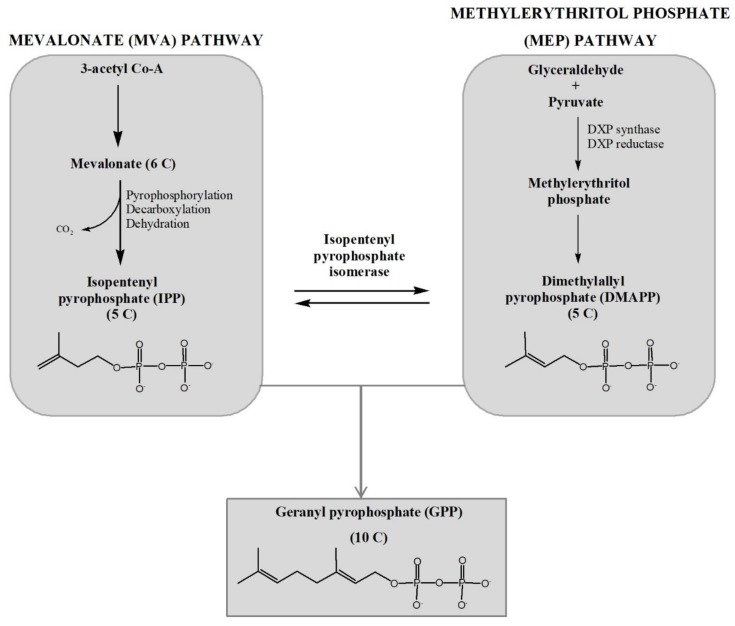
Mevalonate (MVA) and methylerythritol phosphate (MEP) pathway.

Two pathways are known for the biosynthesis of monoterpenes, and the first step includes the formation of the central building blocks of all isoprenoids (known as active isoprenes) isopentenyl pyrophosphate (IPP) and dimethylallyl pyrophosphate (DMAPP). They can be formed by mevalonate (MVA) pathway and methylerythritol phosphate (MEP) pathway which is also known as deoxyxylulose-5-phosphate (DXP) pathway (Figure 1). Each of these pathways can occur in the algae cells. For instance, green algae possess only MEP pathway, while several red algae contain both or only one of the mentioned pathways. Lohr et al. [36] also suggested that further genomic sequencing of species is expected to provide more evidence for distribution of these two pathways. The MVA pathway occurred in the cytosol, while the MEP pathway is localized in the plastids, but both have the same task-to form IPP and DMAPP for the synthesis of the key intermediate geranyl pyrophosphate (GPP) [37].

Even though the MVA pathway was discovered in 1950s in yeasts and animals, it is still considered to be the main route of IPP and DMAPP synthesis. It begins with condensation of three molecules of acetyl-CoA followed by the enzymatically assisted conversions for the formation of IPP, all occurring in the cytosol. Six different enzymes are involved in the formation of IPP with the phosphorylation of mevalonate by mevalonate kinase as the main reaction, while the seventh enzyme is responsible for the generation of DMAPP from IPP [36,38]. 

In contrast to the MVA pathway, the MEP pathway was discovered in 1990s in bacteria and plants. It begins with the reactions between glyceraldehyde-3-phosphate (G3P) and pyruvate and it is capable of producing both IPP and DMAPP as final products due to the presence of the enzyme 4-hydroxy-3-methylbut-2-enyl diphosphate reductase (HDR) [36,39]. IPP is isomerized to DMAPP, which than reacts with IPP within the precursor pool and forms geranyl pyrophosphate (GPP), known as the common precursor for all monoterpenes [40].

### 2.2. Biosynthesis of Cyclic Monoterpenes in Macroalgae

**Figure 2:**
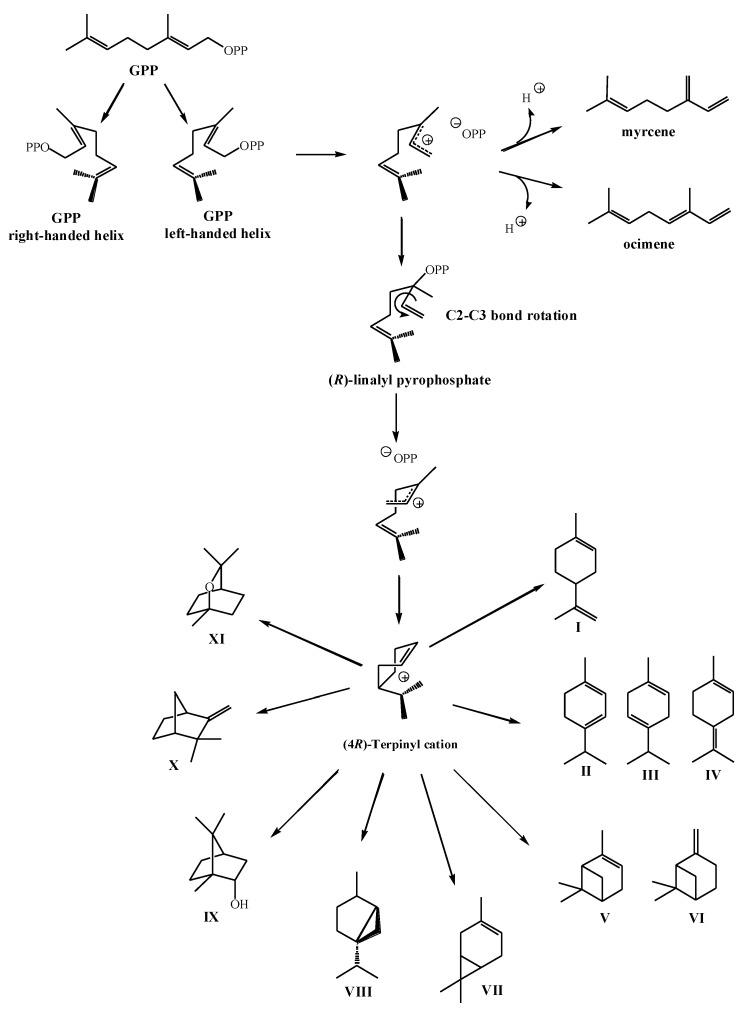
The pathway of monoterpene cyclization through divalent cation-assisted ionization of pyrophosphate group and formation of α-terpinyl cation intermediate (**I**—limonene; **II**—δ-terpinene; **III—**γ-terpinene; **IV**—α-terpinene; **V**—α-pinene; **VI**—β-pinene; **VII**—car-3-ene; **VIII**—sabinene; **IX**—borneol; **X**—camphene; **XI**—1,8-cineol).

Although GPP is considered as the universal precursor for monoterpene biosynthesis, it has limited flexibility for the formation of mono- and bicyclic carbon skeletons. Namely, GPP *trans*-2,3-double bond prevents direct cyclization. Consequently, the enzyme-catalyzed cyclization of GPP and its conversion into tertiary allylic isomer, linalyl pyrophosphate (LPP), occurs in formation of cyclic monoterpenes. The most important step of this mechanism in the algae is the initiation of divalent cation-assisted ionization of the pyrophosphate group which will allow rotation around new single bond and new highly reactive carbocation intermediate is formed, cyclic α-terpinyl cation (Figure 2). α-Terpinyl cation intermediate will undergo several mechanisms such as oxidation, reduction, isomerization, or conjugation, and it will generate a variety of monoterpene cyclic and bicyclic carbon skeletons [41,42]. Figure 2 shows the mechanism of formation of various monoterpene structures from left-handed helical conformer of GPP, but a similar pathway would occur from GPP right-handed helix which would result in the stereochemical antipodes of formed structures.

Wise et al. [41] tested neryl pyrophosphate (NPP), *cis*-isomer of GPP, and LPP as alternate substrates for monoterpene synthesis. The results showed that myrcene synthase exhibited the ability to form cyclic structures from NPP and LPP resulting in different product profiles. Limonene was produced from NPP, while the mix of products was utilized from LPP, such as myrcene, *cis*-ocimene, terpinene, and limonene. Although the enzyme is capable for cyclization of NPP and LPP, it does not catalyze the isomerization of GPP to an intermediate that is able to cyclize. Moreover, they demonstrated for the first time in vitro activity of monoterpene synthase from marine source, which provided the basis for understanding and further exploration of biosynthesis of halogenated monoterpenes.

### 2.3. Biosynthesis of Halogenated Monoterpenes in Macroalgae

**Figure 3:**
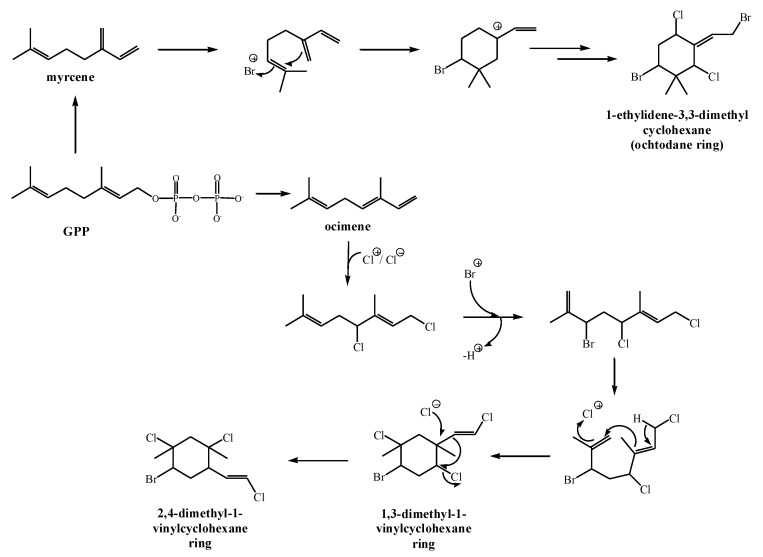
The scheme of cyclization of halogenated monoterpene structures.

Acyclic monoterpenes from macroalgae with bromine or chlorine atoms or both are likely the result of haloperoxidase action on either myrcene or ocimene [33,43]. Bromonium ion-initiated cyclization was proposed to explain the formation of ochtodane ring (l-ethylidene-3,3-dimethylcyclohexane) found in marine monoterpenes [44] from myrcene. Thus, the ring closure is initiated by bromonium attack on C_6_–C_7_ olefin followed by internal addition to the resulting cationic center (Figure 3). Similar reasoning invokes ocimene as the immediate precursor of 1,3-dimethyl-l-vinylcyclohexane ring and 2,4-dimethyl-1-vinylcyclohexane ring [35]. The biogenetic schemes presented in Figure 3 are based on relevant chemical models [45,46,47] and successfully predict halogenation patterns and ring structures observed in the algae.

As mentioned previously, red algae are known for their content of halogenated monoterpenes which are characterized by multiple halogen substitutions [35]. They can be produced as the consequence of conversion of GPP to myrcene with myrcene synthase followed by myrcene halogenation, with the help of haloperoxidases, to mono- or dihalogenated myrcene derivatives [48]. Myrcene and ocimene are immediate precursors for the formation of halogenated cyclic compounds. According to Naylor et al. [35], macroalgae belonging to the genus *Plocamium*, contain ocimene as the common precursor of halogenated monoterpenes, while species from *Portieria* and *Ochtodes* contain myrcene as the precursor of halogenated monoterpenes. However, myrcene is not the only one responsible for the biosynthesis of halogenated monoterpenes since the enzyme co-factors exhibit the key role in their synthesis. Without certain enzyme co-factors, halogenated monoterpenes could not be synthesized. Polzin et al. [49] demonstrated that *Ochtodes secundiramea* (Montagne) M. Howe was not able to synthesize halogenated monoterpenes without the presence of bromide and vanadate which are bromoperoxidase co-factors. This was also confirmed by Polzin and Rorrer [40] when they removed bromide and vanadate from the medium of *O. secundiramea* and consequently bromoperoxidase-catalyzed halogenation did not occur which indicated that bromide and vanadate are required for the biosynthesis of brominated and bromochlorinated monoterpenes in *O. secundiramea*. Furthermore, it was found that *Portieria hornemannii* (Lyngbye) P.C. Silva contains only chlorinated monoterpenes, while *Ochtodes secundiramea* does not, which indicates the variations in halogenation enzyme activity [34]. Moreover, it could not be said for certain that the cyclization of myrcene to ochtodane and other related structures is catalyzed by the enzymes. Fenical [50] showed that the cyclization can occur without the enzyme catalysis in the presence of bromonium ion, while Barahona and Rorrer [34] showed that *P. hornemannii* possess chlorination enzyme which does not require bromonium ion intermediate. Barahona and Rorrer [34] proposed that halogenation of monoterpenes is the consequence of Markovnikov addition of the halogen to the olefinic bond of myrcene which is attacked by the halonium ions. 

## 3. Isolation and Biodiversity of Monoterpenes from Macroalgae

Isolation of monoterpenes from macroalgae belonging to the genus *Plocamium* has been of most interest to various researchers due to the presence of halogenated monoterpenes. The most monoterpenes were isolated from *Plocamium cartilagineum* (Linnaeus) P.S. Dixon collected from different parts of the world (Table 1). 

### 3.1. Comparison of Different Extraction Methods for the Isolation of Monoterpenes

**Figure 4:**
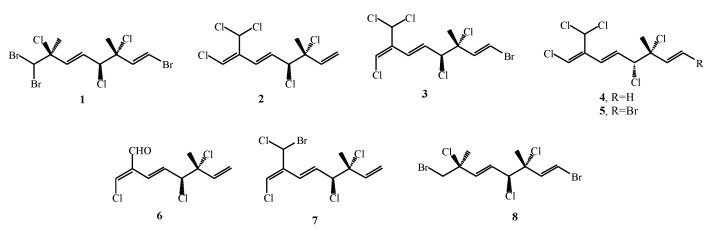
Monoterpenes isolated with SC-CO_2_ extraction from *Plocamium cartilagineum*.

Monoterpenes have been mostly extracted with different solvents, which are presented in Table 1. These methods belong to the group of conventional extraction methods, but Gao and Okuda [51] used supercritical fluid extraction with CO_2_ (SC-CO_2_) for the extraction of monoterpenes (**1–8,** Figure 4) from *P. cartilagineum* collected from two different locations, Santa Cruz and San Diego. They used two reference compounds, (**6**) and (**7**), for determination of the extraction efficiency of SC-CO_2_. SC-CO_2_ with pure CO_2_ and with the addition of co-solvent methanol was investigated as well as the influence of time and pressure on the extraction yield of monoterpenes. The extraction with pure CO_2_ gave lower yields, as well as the incomplete profile of monoterpenes when compared with the extraction with methanol as co-solvent. For obtaining the main monoterpenes from *P. cartilagineum*, additional methanol extraction with a reused algae sample was applied and several monoterpenes were detected in the residual material which remained after SC-CO_2_ with methanol as co-solvent. The yield of the compounds (**6**) and (**7**) was much lower compared to the conventional extraction which can be the consequence of applied process conditions when many unwanted compounds were extracted and interfered with monoterpenes during gas chromatography and mass spectrometry (GC-MS) analysis. Another observation was that the compound (**6**) was unstable and it decomposed during the conventional extraction from *P. cartilagineum* probably due to the presence of an aldehyde group which is sensitive to air oxidation. When comparing conventional and supercritical extraction of monoterpenes, it can be concluded that with supercritical extraction monoterpenes could not be oxidized (their original structure is maintained), while during the conventional extraction these compounds can be oxidized. Namely, the conditions during supercritical extraction are almost oxygen free so the potential of oxidation of the compound (**6**) was reduced. However, the oxidation can also occur during the analysis such as GC [52]. To determine the losses and degradation of certain compounds, external standards with similar or the same chemical structures as the target compound could be used [51,52]. In addition, Gao and Okuda [51] observed that the collection location of the algae showed significant influence on monoterpene profile. The supercritical extraction could selectively extract halogenated monoterpenes from Santa Cruz *P. cartilagineum* when the pressure and time of the extraction were combined. On the other hand, when San Diego *P. cartilagineum* was extracted with supercritical fluid extraction, the selectivity of halogenated monoterpenes was not found.

### 3.2. Monoterpenes Isolated from Different Macroalgae Species

Monoterpenes are divided into cyclic and acyclic groups, with cyclic monoterpenes being mono- or bicyclic. Regular acyclic monoterpenes are head-tail linked (the branched end of one isoprene unit binds to the unbranched end of another isoprene unit). 

**Figure 5:**
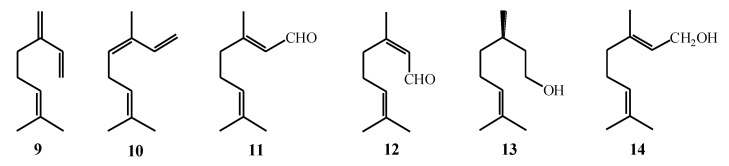
Acyclic monoterpenes.

Figure 5 shows the most significant representatives of acyclic monoterpenes found in the algae: Myrcene (**9**), ocimene (**10**), geranial (**11**), neral (**12**), citronellol (**13**), and geraniol (**14**). The most pleasant odorous compounds found in the algae [53] are included in acyclic group of monoterpenes.

**Figure 6:**
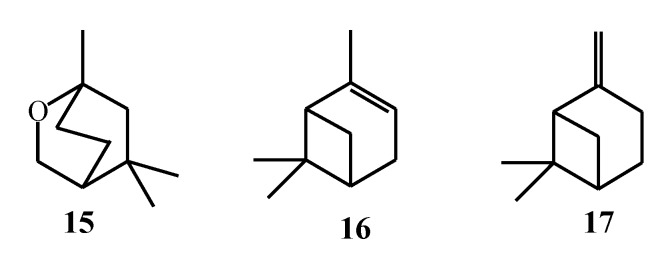
Cyclic monoterpenes.

Monocyclic monoterpenes are usually derived from methyl-isopropyl cyclohexane by its dehydrogenation [54]. 1,8-Cineole (**15**) is the most common monocyclic monoterpene found in the algae [53,55], while α-pinene (**16**) and β-pinene (**17**) are the most common representatives of the bicyclic monoterpenes (Figure 6) [53,56]. 

**Figure 7:**
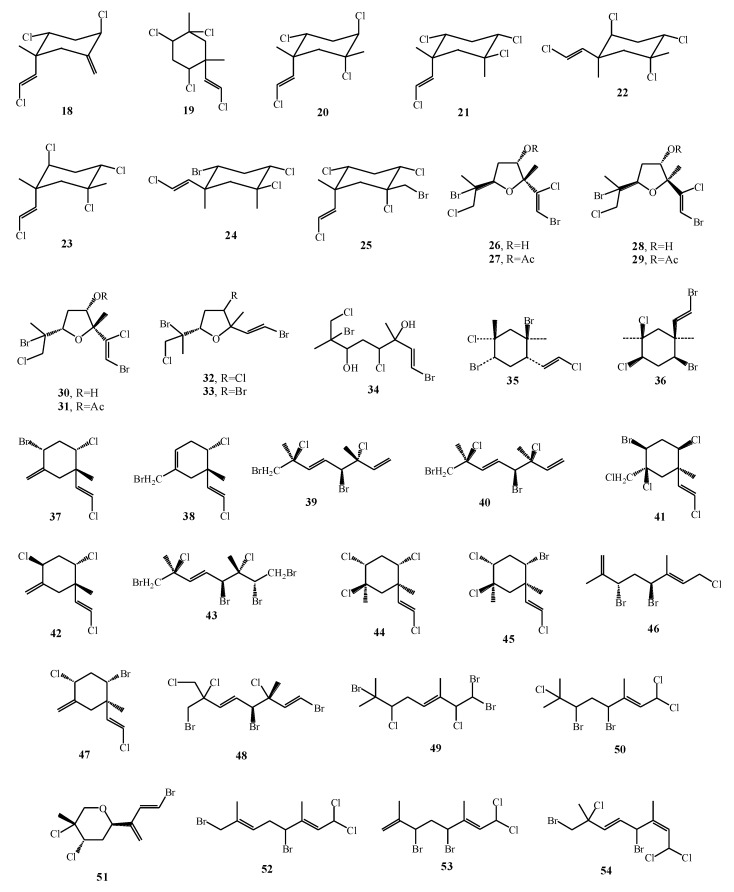

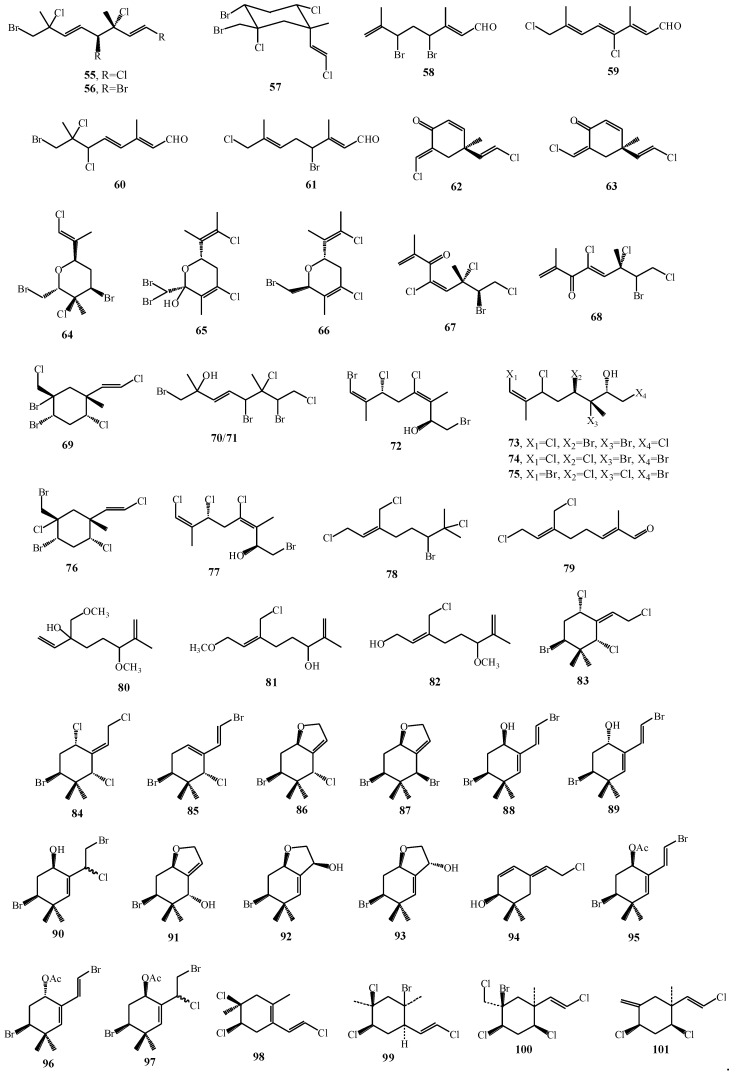
Isolated halogenated monoterpenes from species of genera *Plocamium*, *Portieira*, *Ochtodes*, and *Microcladia*.

Even though monoterpenes isolated from macroalgae have been known for centuries, new emerging methods of their isolation and analysis allowed further research of these compounds with the discovery of many new monoterpenes produced by macroalgae (Figure 7, Table 1). Four new monoterpenes based on 1-(2-chlorovinyl)-2,4,5-trichloro-1,5-dimethylcyclohexane skeleton, (**18**–**21**), and four known cyclic monoterpenes, (**22**–**25**), were isolated and characterized from *P. cartilagineum* collected along the central coast of Chile [57]. Darias et al. [58] collected this alga also from Chilean coast and found that isolated tetrahydrofuran monoterpenes, furoplocamioids A–C (**26**–**31**), contained a chlorobromo vinyl functional group which is unusual among the macroalgae monoterpenes. It is known that monoterpenes isolated from the algae of *Plocamium* genus possess terminal bromovinylic or chlorovinylic system, but the findings of dihalovinyl system enabled the discovery of regiochemistry of new polyhalogenated monoterpenes. Hence, *P. cartilagineum* collected at the same location was investigated for its minor constituents to better understand the formation of oxane ring-containing monoterpenes. Diaz-Marrero et al. [59] described two new tetrahydrofuran halogenated monoterpenes (**32**, **33**), as well as new acyclic polyhalogenated monoterpene (**34**). *P. cartilagineum* collected from the Spanish coast contained two new polyhalogenated acyclic monoterpenes, (**35**) and (**36**) [60]. König, Wright, and Sticher [61] confirmed that the location influenced the secondary metabolites of *P. cartilagineum*, while in 1999, König, Wright, and Linden [16] observed this pattern in *Plocamium hamatum* J. Agardh collected from different regions in Australia and reported the compounds (**37**–**47**).

In 1979, Stierle and Sims [62] studied *P. cartilagineum* collected in Antarctica and found several monoterpenes as minor constituents, while Shilling et al. [63] found that these monoterpenes were present at higher concentrations in the same alga species collected also in Antarctica, but during different seasons. The compounds (**48**–**51**) were discovered as new, undescribed halogenated monoterpenes from *P. cartilagineum*, named anverenes B–E. 

A recent study by Knott et al. [64] stated that red algae of the families *Plocamiaceae* and *Rhizophyllidaceae* developed pathways to oxidize chloride and bromide ions from seawater and incorporate them into monoterpene structures. This resulted in a wide variety of different cyclic and acyclic monoterpenes with multiple halogen atoms, mostly chloride and bromine [27]. Although *P. cartilagineum* was the most studied algae of the genus *Plocamium*, the authors observed that other algae from the same genus also produce halogenated monoterpenes. Knott et al. [17] first reported -CHCl_2_ moiety at C-1 position of three new ocimene-type polyhalogented monoterpenes, plocoralides A–C (**52**–**54**), as well as three known compounds (**55**–**57**) from *Plocamium corallorhiza* (Turner) J.D. Hooker and Harvey collected from the coast of South Africa. Mann et al. [18] observed that *P. corallorhiza* contained unusual moieties attached to monoterpene skeleton. They isolated four new unstable halogenated monoterpene aldehydes (**58**–**61**) which were not detected in the algae collected from the west coast of South Africa, but they were present in the macroalgae from the southeastern coast. According to the similar structure to known compounds, such as plocoralide B, they suggested that the compounds (**58**–**61**) were degradation products formed during the isolation. The aldehyde group at C-1 is usually characteristic to terrestrial monoterpenes but they were first to report this functional group in marine halogenated monoterpenes. Knott et al. [64] described the isolation and characterization of two novel monoterpenes, halogenated cyclohexanone derivatives, plaxenone A and plaxenone B (**62**, **63**) from *Plocamium maxillosum* (Poiret) J.V. Lamouroux. A recent study on *Plocamium angustum* (J. Agardh) J.D. Hooker and Harvey from New Zealand revealed the presence of new polyhalogenated monoterpene with tetrahydropyran ring, costatone C (**64**), isolated from this species for the first time [65], while costatone A (**65**) [66] and costatone B (**66**) [67] were isolated earlier from different *Plocamium costatum* (C. Agardh) J.D. Hooker and Harvey samples collected in South Australia. 

Several monoterpenes are very unstable and during their isolation degradation can occur. During the last few years, a new hyphenated technique, high-performance liquid chromatography–nuclear magnetic resonance (HPLC-NMR), has been employed for obtaining the chemical profile of the genus *Plocamium*. This technique is non-destructive and all of the isolated compounds are fully recovered. Dias and Urban [68] successfully used this technique to obtain phytochemical profile of *Plocamium mertensii* (Greville) Harvey which represents the first application of this technique on marine algae. While they obtained the mixture of compounds and isolated other compounds besides monoterpenes, Timmers et al. [69] used this technique to obtain and identify two pure monoterpene compounds. First, they obtained an already known compound, plocamenone (**67**), from *Plocamium angustum* and then they identified its unstable double bond isomer isoplocamenone (**68**) and concluded that this is a very valuable method for the analysis of unstable compounds. On the other hand, Motti et al. [67] used the HPLC-UV-MS-SPE-NMR technique for determination of halogenated monoterpenes from *P. costatum* and *P. hamatum.* This technique is relatively new and it combines traditional and hyphenated chromatographic techniques which employs solid-phase extraction (SPE) as an interface between traditional chromatography, ultraviolet spectroscopy (UV), liquid chromatography-mass spectrometry (LC-MS) and nuclear magnetic resonance (NMR). It has the advantage over HPLC-NMR technique because the analytes trapped on SPE cartridge are eluted with minimal volumes of solvent directly into NMR flow-probe. The results indicated new halogenated monoterpenes including the compounds (**66**, **69**–**75**), and two known halogenated monoterpenes (**76**) and (**77**). The compound (**70**) was isolated as the mixture with compound (**71**) at the ratio of 2:1. These two compounds showed similar ^1^H NMR spectrum with only very slight differences in their chemical shifts (Δδ_H_ 0.1), indicating that these two compounds are stereoisomers. However, their absolute configuration was unassigned.

Similar as genus *Plocamium*, genus *Portieria* showed differentiation of monoterpene content depending on the collection location, as was shown by Wright et al. [70] during the isolation of compounds (**78**–**82**) from *Portieria hornemannii* collected in Australia. Gunatilaka et al. [71] isolated two new regioisomeric tetrahalogenated mnonoterpenes, named apakaochtodene A (**83**) and apakaochtodene B (**84**), from *P. hornemannii* collected from different reef sites on Guam with different ratios.

Even though most of the studies are reported on *Plocamium* and *Portieria* genera (as the most promising producers of both cyclic and acyclic halogenated monoterpenes), genus *Ochtodes* also contained halogenated monoterpenes [40,48]. Paul et al. [32] isolated 13 new cyclic monoterpenes (**85**–**97**) from red alga *Ochtodes crockeri* Setchell and N.L. Gardner from the Galapagos Islands. In 1976, Crews et al. [72] observed that halogenated monoterpenes found in *Microcladia* differ in the composition between different collection locations. They compared *Microcladia* with *Plocamium* species [73] with respect to the similarity of monoterpenes profile. Based on the results they concluded that the unique pattern of monoterpene structures for differentiation of algae species cannot be applied because the species belonging to *Microcladia* and *Plocamium* can produce similar or the same monoterpenes. Even though they were collected from different locations, plocamene-B (**98**), plocamene-C (**99**), violacene (**100**), and plocamene-D (**101**) were found in all species of the both genera *Microcladia* and *Plocamium*. The compounds present in both genera were related to dechlorobromination and dehydrobromination, while bromine appeared to be the most often incorporated into monoterpenes.

## 4. Bioactivity of Monoterpenes in Macroalgae

Scientists have shown interest for the bioactivity of monoterpenes isolated exclusively from red algae, with genus *Plocamium* and *Portieria* as dominating. El Gamal [7] represented red algae as the main source of monoterpenes with cytotoxic activity. As demonstrated in Table 2, anticancer activity of monoterpenes against different tumor cell lines, including esophageal [74], brain, renal, colon [75], and cervical cancer [76], was the main bioactivity of novel monoterpenes as well as those that were known before. 

**Figure 8:**
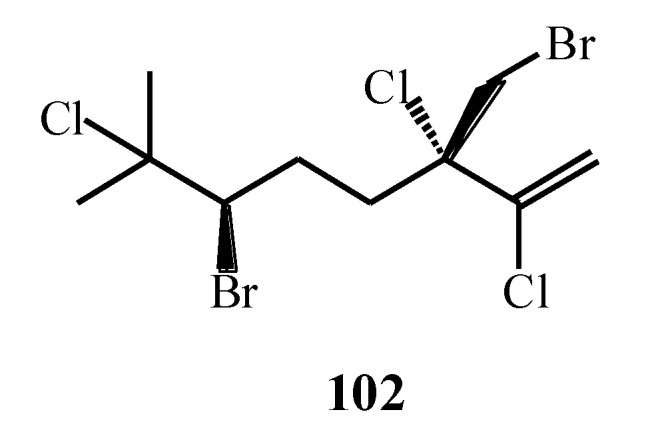
Chemical structure of halomon.

Among all of isolated monoterpenes, halomon is the best-studied halogenated monoterpene isolated from *Portieria hornemannii* whose structure is presented in Figure 8. It was firstly isolated and elucidated by Fuller et al. [75]. Halomon (**102**) was tested for cytotoxicity against human brain, colon, and renal tumor cell lines and the results indicated high cytoxicity against mentioned cell lines, while lower activity was observed against leukemia and melanoma cell lines. Interestingly, *P. hornemannii* collected from different locations showed the absence of halomon, but new monocyclic monoterpene (**103**) with lower cytotoxic activity was observed. *Plocamium hamatum* collected from six different locations showed significant difference within the content of secondary metabolites which led to different cytotoxic results [16]. 

**Figure 9:**
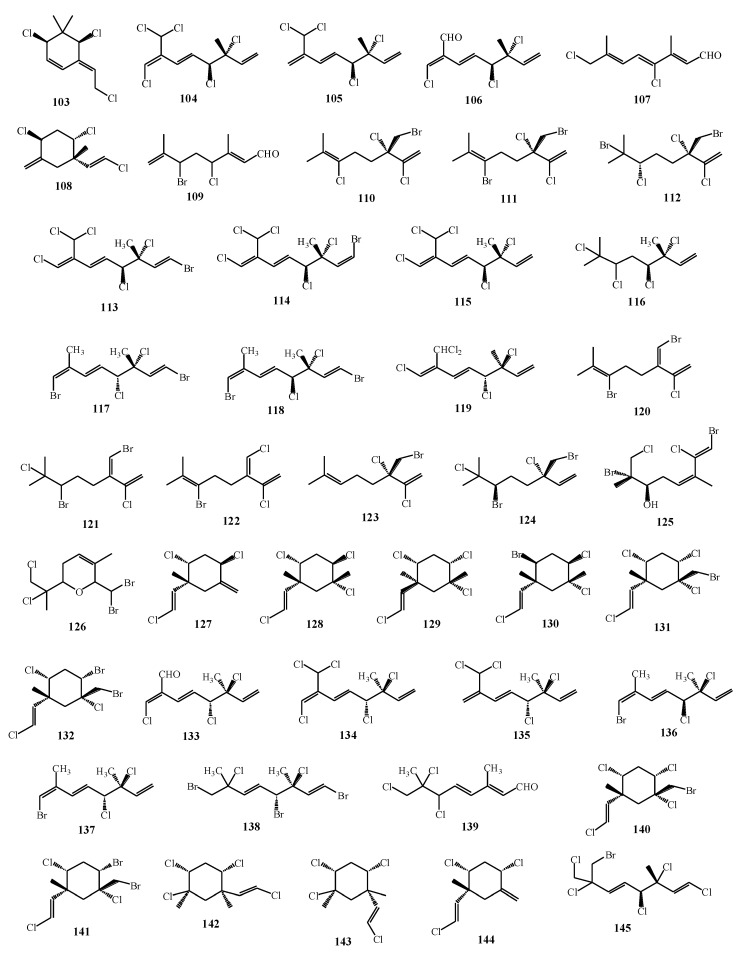
Bioactive halogenated monoterpenes isolated from macroalgae.

The structural diversity among halogenated monoterpenes (Figure 9) has been shown as the important factor for bioactivity intensity. De la Mare et al. [77] isolated monoterpene compounds (**54**, **55**, **58**, **60**, **104**–**109**) and observed that all of them contained from two to five halogen moieties, specifically Cl and/or Br. They selected the molecules whose terpene backbone was identical, but the halogen substitution varied between the compounds. The results indicated that a higher number of halogen atoms resulted in higher anticancer activity against breast cancer cells. Five halogen atoms were found among the most toxic compounds, while non-toxic compounds contained three and four halogens. Hence, the most active against breast cancer cells was the compound which contained only Cl atoms, without Br. When halomon-related halogenated monoterpenes (**110**–**112**) were isolated and tested for cytotoxicity, the results showed that the halogen at position C-6 was essential for the certain activity [78]. 

Due to relatively simple structures of the algal compounds, they can be used for novel chemotherapeutics. Even though their activity is lower than commercially used chemotherapeutics, their structures, along with their differential toxicity, promise novel mechanisms of action against cancer cell lines. Further studies should be performed to develop further modifications for the enhancement of their activity. However, Antunes et al. [74] observed that the cytotoxic activity of halogenated monoterpenes **(5**, **113**–**118**) from *Plocamium suhrii* Kützing and *Plocamium cornutum* (Turner) Harvey was higher when compared to the known anticancer drug *cis*-platin. Knott et al. [17] showed that plocoralides A–C (**52**–**57**), polyhalogenated monoterpenes from *Plocamium corallorhiza*, exhibited good activity against human esophageal cancer cell lines when compared to the commonly used *cis*-platin. It can be observed that the genus *Plocamium* produces a great variety of polyhalogenated monoterpenes with various anticancer activities (Table 2). Sabry et al. [79] isolated one new halogenated monoterpene (**119**) from *P. cartilagineum* collected from South Africa. It showed good cytotoxic activity against the cells of human lung cancer and mouse neuro-2a cell lines. Halomon **102** along with the compounds **120**–**124** isolated from *Portieria hornemannii* were good inhibitors against DNA methytransferase-1 isoform, which represents the enzyme responsible for tumor growth [80]. De Ines et al. [76] showed that halogenated monoterpenes could have selective activity against certain cancer cell lines. Among isolated compounds (**30**, **125**, **126**, **127**–**132**) from *P. cartilagineum,* the most potent activity exhibited the compounds **30**, **125**, **126**, and **128** with notably selective cytotoxicity against colon and cervical adenocarcinoma cells. Halogenated monoterpenes **62** and **63** isolated from *Plocamium maxillosum* were tested against MDA-MB-231 metastatic breast carcinoma cell line and showed moderate antiproliferative activity [64]. This level of activity of both of the compounds **62** and **63** is comparable with the previously reported activities of halogenated monoterpenes (**54**, **55**, **58**, **60**, **104**–**109**) against the same cancer cell lines [77]. 

Geographical variation, except on anticancer activity, showed also an influence on antiplasmodial activity of halogenated monoterpenes isolated from *Plocamium cornutum* [81]. Afolayan et al. [81] also emphasized the importance of dichloromethyl moiety at position 7 when considering the higher antiplasmodial activity of halogenated monoterpenes. They observed that two novel compounds **134** and **135** containing 7-dichloromethyl moiety showed significantly higher activity toward strains of *Plasmodium falciparum* compared with already known compounds **53**, **58**, **133**–**135**, **138**, and **139**. Furthermore, two novel cyclohexane polyhalogenated monoterpenes **140** and **141** and furoplocamioid C (**30**) reported earlier [58] were very efficient repellents and antifeedants, as well as selective insect cell toxicants depending on the number of halogenated substituents, as well as halogen type. Strong antifeedant activity was observed when five halogen substituents were present in monoterpene skeleton, but when compared to six halogen substituents the activity was significantly decreased. The substitution of Br atom showed higher activity of the molecule when compared with C-l atom [82]. 

According to Watanabe et al. [83], red algae belonging to the genus *Plocamium* exhibit strong insecticidal activities (Table 2). They studied two polyhalogented monoterpenes, aplysiaterpenoid A (**142**) and telfairine (**143**), isolated from *Plocamium telfairiae* (W.J. Hooker and Harvey) Harvey ex Kützing and tested their insecticidal activities against *Blatella germanica* and *Anopheles gambiae*. Aplysiaterpenoid A (**142**) and telfairine (**143**) possess cyclodiene-type insecticidal mode of action due to the orientations of the sterically bulky regions and electronegative centers in monoterpenes. In that way, the geometrical requirement for the interaction of monoterpenes with picrotoxinin receptor in tested insects was satisfied, while large volumes of both monoterpenes led to the loose fitting into the receptor. Monoterpenes isolated from *Plocamium cartilagineum* also showed insecticidal activities. Specifically, the compounds **18**–**25** were tested against several species of insects among which violacene (**19**) showed the highest activity against *Macrosteles facifrons* [57]. Rovirosa et al. [84] also isolated monoterpenes from *Plocamium cartilagineum* collected from Antarctica. The compound **144** showed the most potent activity against *Heliothis virescens*, while the compound **145** showed a mild activity against *Diabrotica undecimpunctata*. 

On the other hand, some authors tested the extracts obtained with different methods from various macroalgae species. Machado et al. [85] showed that the obtained extracts with the highest concentration of monoterpenes exhibited acetylcholinesterase inhibition (AChEI). According to the presence of halogenated monoterpenes, *Ochtodes secundiramea* showed the most potential for AChEI activity. Further studies are necessary for understanding the activity mechanism. 

## 5. Conclusions

The structural diversity of macroalgal monoterpenes is influenced by the habitat of macroalgae (different light exposure and water temperature), as well as the season of their collection. Among the macroalgae belonging to the same genus, there are variations in terms of monoterpene profile and in the ratios of present monoterpenes, while some of monoterpenes are even absent. Even though some species (as those belonging to the genus *Plocamium*) were very well investigated in the past, the diversity of monoterpenes and the application of new hyphenated techniques lead to the discovery of new and yet unknown monoterpenes in the last few years. In this paper, the total of 136 different structures of monoterpenes isolated from the genera *Plocamium, Protieria, Ochtodes*, and *Microcladia* were shown along with nine structures of commercially known monoterpens which can be found in other plants, as well as in the algae. According to the literature presented, red algae from the genus *Plocamium* are the largest source of acyclic and cyclic monoterpenes. Moreover, with more than 100 isolated monoterpenes, *Plocamium* species are one of the most important macroalgal sources of these compounds. Halogenation is characteristic for macroalgal monoterpenes due to the capability of algae to synthesize diverse halogenated monoterpenes with the help of haloperoxidases present in their cells and/or Markovnikov addition of the halogen on the precursor which is attacked by the halonium ions. Macroalgal monoterpenes are mostly halogenated with one or more chlorine and/or bromine atoms in their structure. 

Numerous studies have shown that monoterpenes isolated from macroalgae exhibit anticancer, insecticidal and antiplasmodial activity, while the extracts rich in monoterpenes exhibited acetylcholinesterase inhibition, but further elucidation about the activity mechanisms should be performed. It was shown that the structure of monoterpenes affects the bioactivity intensity. Higher halogen substitution leads to the better cytotoxicity and five halogen atoms were found as optimal. Hence, halogenated monoterpenes with only C-l atoms showed higher activity.

Due to the fact that the marine biodiversity is greater than terrestrial, the discovery of new monoterpenes is expected to increase.

## Figures and Tables

**Table 1:** The methods of isolation of halogenated monoterpenes from different macroalgal species.

Macroalgae Species	Isolated Monoterpenes	Extraction Solvent	Analytical Method	References
**Genus *Plocamium***				
*Plocamium cartilagineum*	**1**–**8**	Conventional extraction with MeOH and EtOAc; SC–CO_2_ extraction (pure CO_2_ and with 10 % MeOH as co-solvent)	GC–MS, NMR	[51]
*Plocamium cartilagineum*	**18**–**25**	CHCl_3_ and EtOH	NMR	[57]
*Plocamium cartilagineum*	**26**–**31**	EtOAc–CH_2_Cl_2_-hexane	HPLC, NMR	[58]
*Plocamium cartilagineum*	**32**–**34**	hexane/EtOAc/CH_2_Cl_2_/MeOH	HPLC, NMR	[59]
*Plocamium cartilagineum*	**35, 36**	Et_2_O	NMR	[60]
*Plocamium hamatum*	**37**–**47**	CH_2_Cl_2_	NMR	[16]
*Plocamium cartilagineum*	**48**–**51**	CH_2_Cl_2_/H_2_O	GC–MS, HPLC, NMR	[63]
*Plocamium corallorhiza*	**52**–**57**	MeOH and CH_2_Cl_2_	NMR	[17]
*Plocamium corallorhiza*	**58**–**61**	CH_2_Cl_2_–MeOH	NMR	[18]
*Plocamium maxillosum*	**62, 63**	CH_2_Cl_2_–MeOH	NMR	[64]
*Plocamium angustum*	**64**	MeOH	NMR, HPLC	[65]
*Plocamium costatum*	**65**	Hexane	NMR	[66]
*Plocamium angustum*	**67, 68**	CH_2_Cl_2_–MeOH	HPLC–NMR	[69]
*Plocamium hamatum, Plocamium costatum*	**66, 69**–**77**	CH_2_Cl_2_–MeOH	HPLC–UV–MS–SPE–NMR	[67]
**Genus *Portieria***				
*Portieria hornemannii*	**78**–**82**	MeOH:CH_2_Cl_2_	NMR	[70]
*Portieria hornemannii*	**83**, **84**	CH_2_Cl_2_/MeOH	HPLC, NMR	[71]
**Genera *Ochtodes* and *Microcladia***				
*Ochtodes crockeri*	**85**–**97**	CHCl_3_:MeOH	HPLC	[32]
*Microcladia coulteri,* *M. borealis,* *M. californica*	**98**–**101**	CHCI_3_, CH_2_Cl_2_ and EtOH, CHCl_3_ and EtOH	GC–MS, NMR	[72]

**Table 2:** Isolated monoterpenes from different macroalgal species and their bioactivity.

Macroalgae Species	Isolated Monoterpenes	References
**Anticancer activity**		
*Portieria hornemannii*	**102, 103**	[75]
*Plocamium corallorhiza, Plocamium cornutum*	**54, 55, 58, 60, 104–109**	[77]
*Portieria hornemannii*	**110–112**	[78]
*Polcamium suhrii, Plocamium cornutum*	**5, 113–118**	[74]
*Plocamium corallorhiza*	**52–57**	[17]
*Plocamium cartilagineum*	**119**	[79]
*Portieria hornemannii*	**102, 120–124**	[80]
*Plocamium cartilagineum*	**30, 125, 126, 127–132**	[76]
*Plocamium maxillosum*	**62, 63**	[64]
**Antiplasmodial (antimalarial) activity**		
*Plocamium cornutum*	**53, 58, 133–139**	[81]
**Insecticidal activity**		
*Plocamium cartilagineum, Pantineura plocamioides*	**26, 27, 30, 31, 140, 141**	[82]
*Plocamium telfairiae*	**142, 143**	[83]
*Plocamium cartilagineum*	**18–25**	[16]
*Plocamium cartilagineum*	**144, 145**	[84]
